# Supplementary material for: Maternal adverse childhood experiences and perinatal outcomes: A retrospective inceptive cohort study
Source: PLoS One. 2025 Nov 26;20(11):e0333366. doi: 10.1371/journal.pone.0333366 (PMC12654885; doi:10.1371/journal.pone.0333366)
Supplement: Attachment — Submitted filename: Rebuttal letter PLOS ONE 2025-05-13.docx [file pone.0333366.s003.docx]

Re: PONE-D-24-51497

Dear Editor,

Thank you and the reviewers for your encouraging feedback!

Please find our responses to the reviewers’ comments below. The changes are marked with yellow underline.

Reviewer #1

1. An error with the attrition reported.

Answer: On page 6, 2^nd^ paragraph, the figure regarding “Q2 received Q3” is changed to “3389”.

2. Statistics for group differences for Q1 and Q3.

Answer: On page 7, 1st paragraph, statistics highlighting group differences between women responding to Q3 and those answering Q1 only have been included.

3. Control for previous pregnancies/complications? Empirically explored or listed as a limitation.

Answer: We reference the response given under item 5.

4. “Other unmeasured confounders may also have biased our results” Provide examples.

Answer: On page 28, in the 2^nd^ paragraph, we have added ”as the mothers’ parents’ experiences of ACEs”.

5. Selection of confounders? DAG? Why not control for smoking, alcohol use, and BMI?

Answer: On page 11, 1st paragraph, the following text is added:

“The selection of confounders followed established principles, such as pretreatment criterion and causal diagram. Applying the pretreatment criterion, parental origin and socioeconomic status were identified as variables that precede exposure to adverse childhood experiences (ACEs). Using a causal diagram and the “back-door path criterion,” these two factors were further confirmed as key for mitigating confounding bias. The diagram also suggested that variables such as a new partner, stress during pregnancy, alcohol consumption, smoking habits, BMI, education level, age, parity, and pre-pregnancy hypertension act as mediators. Adjusting for these mediators could introduce bias. Given the absence of data on parental socioeconomic status, origin was selected as the confounder for adjustment in the regression analyses.

A sensitivity analysis was conducted that included an alternative causality model based on other assumptions about the complexity among potential confounders as part of a chain of confounders or mediators. In this model, adjustments were made for age, high educational level, smoking habits, alcohol habits, body mass index and number of previous pregnancies in addition to adjustments in the causal diagram model.”

On page 11, 3^rd^ paragraph we have added information of an additional sensitivity analysis:

“In addition, a sensitivity analysis was conducted to evaluate the robustness of the association to potential unmeasured and uncontrolled confounding, using the E-value calculation, by the formula E-value=*OR + sqrt (OR x (OR-1)).”*

On page 20, 3^rd^ paragraph we have added “The calculated E-value for the adjusted odds ratio for preeclampsia of 4.21 was 7.9.”

On page 28, 3^rd^ paragraph we have added the following text:

“To address potential confounding, a sensitivity analysis was conducted. For preeclampsia, the observed odds ratio of 4.21 could theoretically be explained by a confounder with a stron association with both the exposure and outcome, featuring an odds ratio of 7.9 times greater than the adjusted odds ratio. However, weaker confounding associations would not suffice. Therefore, relatively strong confounding relationships would be necessary to fully explain the observed exposure-outcome association with an odds ratio of 4.21. Second, an alternative causality model based on other assumptions about the complexity among potential confounders gave similar adjusted odds ratios as the regression with a minimal set of confounders.”

6. DAG is unclear

Answer: Referencing the response provided under item 5 clarifies the DAG.

7. Required copy editing

Answer: The manuscript has undergone a thorough linguistic review. Hopefully, this has enhanced the reader's comprehension.

Reviewer #2

1. Introduction, ACE outcomes to be specified.

Answer: On page 5, 1^st^ paragraph references are added to the specific adverse health outcomes.

2. The acronym ACEs would be used.

Answer: Changed to ACEs throughout where appropriate.

3. Abstract. Clarify and revise these sentences in acknowledgement of the previous literature.

Answer: In the Abstract Introduction: “scarce” is changed to “remains limited, and the effect of cumulative ACEs on perinatal outcomes is unknown”.

In addition, on page 5, 3^rd^ paragraph in the Introduction we have changed the wording to: “However, research on the impact of multiple ACEs on perinatal outcomes remains limited.”

4. Attrition reported.

Answer: We refer to the response provided to Reviewer #1 under item 1. Adjusting the denominators simplifies tracking attrition, thereby reducing the need for a flowchart.

5. The results of the differences among women answering Q3 and Q1 only, should include significance testing.

Answer: On page 7, 1^st^ paragraph, the actual proportions and p-values have been incorporated.

6. Who conducted translation process, bilingual?

Answer: On page 7, 2^nd^ paragraph the following text is added:

“This process involved three translators: one native English speaker with Swedish as a second language, and two native Swedish speakers with English as their second language. The translated questionnaire was then compared to the original to ensure accuracy.”

7. What do the authors mean with “until any of the criteria described in details elsewhere was true.

Answer: On page 8, 1^st^ paragraph this sentence is added: “Each ACE category was initially coded as not experienced unless any of the criteria, described in an earlier study by Andersson et al in 2021 (3) were met”.

8. Obstetric complications. What data is considered in “other”

Answer: On page 8, 2^nd^ paragraph the following text is added:

“The option “other” provided no further details and was selected by 93 of 1,235 women (7.5%).”

9. Please, define “SEK”

Answer: On page 10, 2^nd^ paragraph: “Swedish krona” is added.

10. An appendix table for all variables.

Answer: In the Methods section from page 7, 3^rd^ paragraph to page 10, 4^th^ paragraph, we have included an explanation of how the various questions were formulated and operationalized.

11. Avoid introducing bias.

Answer: We reference the response provided to Reviewer #1 under item 5.

12. The ethics statement. What was the process used to obtain informed consent and documented?

Answer: On page 13, 1^st^ paragraph, the process for obtaining informed consent has been thoroughly detailed.

13. The results section is too brief and not comprehensive. Table 4

Answer: The results are enhanced with statistical figures where relevant and include an additional sentence referring to Table 4.

14. There is no interpretation of the OR in the discussion. Avoid “risk” and “rates”.

Answer: On page 26, the wording of the 1^st^ paragraph has been revised according to your suggestion.

15. Control also for age, education, income and relationship status, as common potential confounders.

Answer: We reference the response provided to Reviewer #1 under item 5.

16. Since the impacts on placental development were not assessed, revise the sentence.

Answer: On page 26, 1^st^ paragraph, the word “indicates” is changed to “suggest”.

17. Revise sentences:

Answer: On page 6, 1^st^ paragraph, the sentence is changed according to the suggestion from the reviewer.

18. Unclear sentence

Answer: On page 6, 2^nd^ paragraph, the sentence has been revised for clarity and comprehensibility based on reviewers’ suggestions

19. Miss of a word.

Answer: On page 6, 3^rd^ paragraph, the word “status” is added to the sentence.

On behalf of the entire group of authors

Kind regards,

Per Kristiansson

Corresponding author
